# Reply to Comment by Steinberger on ‘Will Earth's next supercontinent assemble through the closure of the Pacific Ocean?’

**DOI:** 10.1093/nsr/nwac254

**Published:** 2022-11-09

**Authors:** Chuan Huang, Zheng-Xiang Li, Nan Zhang

**Affiliations:** Earth Dynamics Research Group, The Institute for Geoscience Research (TIGeR), School of Earth and Planetary Sciences, Curtin University, Australia; Key Laboratory of Orogenic Belts and Crustal Evolution, School of Earth and Space Sciences, Peking University, China; Earth Dynamics Research Group, The Institute for Geoscience Research (TIGeR), School of Earth and Planetary Sciences, Curtin University, Australia; Key Laboratory of Orogenic Belts and Crustal Evolution, School of Earth and Space Sciences, Peking University, China

Geodynamic modeling of the supercontinent cycle crosses the fields of geology, geophysics and computational science. We thus welcome the comment by Prof. Steinberger from the numerical geodynamics point of view, which gives us the opportunity to clarify some of the concepts, explain further our model approach and necessary simplifications, and point out major ambiguities that require further work.

Prof. Steinberger's first criticism revolves around the secular change in the strength of the oceanic lithosphere. There are two points to be made here. First, geodynamic modeling work consistently points out the need for a weaker oceanic lithosphere than that of experimental results, in order to generate plate-like behavior in the modeling [1–5]. This inconsistency might reflect the importance of the time factor in lithospheric deformation, which is analogous with the ductile-style deformation of brittle supracrustal rocks during orogenic events over millions of years.

In terms of the secular change in the strength of the oceanic lithosphere, Prof. Steinberger's view appears to be strongly influenced firstly by the traditional pure thermal dynamic view, which oversimplified the top thermal boundary layer (i.e. the conductive layer) as the lithosphere, resulting in a thicker lithosphere as the mantle cooled; and secondly by his belief in the applicability of the so-called ‘Christmas tree’ lithospheric strength profiles to the oceanic lithosphere. We explain below why both assumptions might be incorrect here.

It is now widely recognized that the degree of mantle partial melting along mid-ocean ridges plays a more important role in the thickness and strength of the oceanic lithosphere; with a hotter mantle, partial melting and dehydration stiffening [6] occurs to a greater depth, which not only leaves a thicker depleted mantle lithosphere [7,8], but also a thicker oceanic crust as more melts are being generated [7,9,10]. Following the oceanic lithospheric strength profiles, as in Figs S7d and S8 in the Supplementary Data of our paper [11], the oceanic lithosphere would become weaker as the Earth cooled, as both the crust and the mantle lithosphere became thinner with time. In addition, according to van Keken *et al.* [12], increased mantle hydration with time by subduction may also weaken the oceanic lithosphere.

The typical Christmas-tree-like lithospheric strength profiles mentioned by Prof. Steinberger are generally applicable to the continental lithosphere only, where the lower crust is commonly a weak layer due to weakening of its felsic components under increased temperature [13]. Such a weakening effect is not applicable to the mafic and much thinner oceanic crust and its mantle lithosphere (see Figs S7d and S8 in the Supplementary Data of our paper [11]).

Our Figs S7d and S8 [11] also show that the strength of the oceanic lithosphere rests mostly within its top 0–50 km, where creep flow has not taken over the deformation of the mantle rocks, and the crust plays a significant role, as its strength is not as sensitive to temperature as the mantle rocks [14].

Our model [11] suggests that the lowering of oceanic lithospheric strength with time appears to play a controlling role in how a supercontinent assembles, and that no introversion is possible after the Neoproterozoic supercontinent Rodinia. This is consistent with the argument that the last supercontinent Pangea assembled through extroversion [15], and it predicts that the next supercontinent will form via the closing of the Pacific Ocean.

The other criticism by Prof. Steinberger was about the possible effect of the lithospheric weak zones, which we introduced to our model in order to initiate oceanic subduction, on our overall model results. While the mechanism(s) of subduction initiation is by itself a fundamental science question that is currently hotly debated [16], in our model we introduced weak zones along ocean/continent boundaries when the oceanic crust along such boundaries became older than 200 million years. This simplified model design is necessary to generate plate-like behavior for the modeled lithosphere, and is consistent with real-world observations of how long the oceanic lithosphere can survive. Our model results are not controlled by this model design because, first, subduction does initiate in internal oceans and introversion does occur in some of our models (e.g. Fig. S1 and other figures; [11]); and second, as explained in section ‘Possible effects of the lower-mantle thermo-chemical layer, mantle internal heating and lithospheric weak zones’, we did vary the viscosity of the weak zones by an order of magnitude (0.01 vs. 0.1) in our model and found that it does not change the model results with regard to how a supercontinent is assembled [11].

Prof. Steinberger also queried whether our modeling results might remain the same had we had more ridge-like features along zones of oceanic spreading. This is a fair question and we are now in the process of generating mid-ocean ridge-like features in our model. Our current model failed to generate such features, possibly because we did not consider the viscosity drop caused by partial melting in the oceanic lithosphere. However, as we explained in section ‘Possible effect of diffusive mid-ocean ridges in the modeling’ of our paper [11], this deficiency has little effect on the age of the oceanic lithosphere close to the continental margins within the internal ocean(s), as the ages there are defined by the continental break-up time. As such, creating more ridge-like features would not change the timing of such regions becoming weak zones or their subduction initiations, and thus the overall model results.
